# Atypical Chemokine Receptors and Their Roles in the Resolution of the Inflammatory Response

**DOI:** 10.3389/fimmu.2016.00224

**Published:** 2016-06-10

**Authors:** Raffaella Bonecchi, Gerard J. Graham

**Affiliations:** ^1^Humanitas Clinical and Research Center, Rozzano, Italy; ^2^Department of Biomedical Sciences, Humanitas University, Rozzano, Italy; ^3^Chemokine Research Group, Institute of Infection, Immunity and Inflammation, University of Glasgow, Glasgow, UK

**Keywords:** chemokines, immunity, inflammation, scavenging, atypical receptors

## Abstract

Chemokines and their receptors are key mediators of the inflammatory process regulating leukocyte extravasation and directional migration into inflamed and infected tissues. The control of chemokine availability within inflamed tissues is necessary to attain a resolving environment and when this fails chronic inflammation ensues. Accordingly, vertebrates have adopted a number of mechanisms for removing chemokines from inflamed sites to help precipitate resolution. Over the past 15 years, it has become apparent that essential players in this process are the members of the atypical chemokine receptor (ACKR) family. Broadly speaking, this family is expressed on stromal cell types and scavenges chemokines to either limit their spatial availability or to remove them from *in vivo* sites. Here, we provide a brief review of these ACKRs and discuss their involvement in the resolution of inflammatory responses and the therapeutic implications of our current knowledge.

## Introduction

An effective inflammatory response requires carefully regulated initiation, maintenance, and resolution phases (1). Inflammation is characterized by a stepwise recruitment of leukocytes, with neutrophils typically being the first recruited cellular population, followed by macrophages and lymphocytes. The precise molecular control of inflammation has not yet been fully worked out, although it is clear that the primary regulators of *in vivo* leukocyte migration to inflamed tissue sites are the chemokines, or chemotactic cytokines (2). Chemokines are members of a large family of proteins defined by the presence of a conserved cysteine motif in their mature protein sequences. Chemokines are divided into CC, CXC, XC, and CX3C subfamilies according to the specific nature of the cysteine motif (3). Chemokines are exclusive to vertebrates (4), and the primordial chemokine is almost certainly CXCL12, which was evolved to regulate stem cell migration during embryogenesis. From this one ancestral gene, the family has expanded to the point at which mammals have around 45 different chemokines, which are involved, in sometimes extremely complex and subtle ways, in regulating immune and inflammatory cell migration *in vivo*. Chemokines can be broadly defined as being either inflammatory or homeostatic according to the contexts in which they function (3, 5). Inflammatory chemokines are not normally expressed at significant levels but are induced extremely rapidly following tissue insult, or infection, and serve to recruit inflammatory leukocytes to any compromised body site. In all likelihood, all cells are capable of producing inflammatory chemokines and thus initiating inflammation. In contrast, homeostatic chemokines are involved in the basal recruitment of cells involved in immune responses, and these control much more specific cellular navigation processes.

Chemokines interact with their target cells by binding to receptors belonging to the 7-transmembrane-spanning family of G protein-coupled receptors (6). Thus far, 10 receptors for CC chemokines, 7 for CXC chemokines, and single receptors for the XC and CX3C chemokines have been identified. Again, these receptors can be defined as being either inflammatory or homeostatic according to the class of chemokines they bind. One complex feature of chemokine receptors, particularly those involved in regulating inflammatory leukocyte migration, is that they display promiscuous ligand binding. In addition, individual chemokines can bind to more than one receptor and individual receptors are expressed on numerous different leukocyte cell types (6). Moreover, the formation of receptor dimers and oligomers at the cell surface can modify their chemokine binding and signaling activity, further complicating biology (7). This biological complexity, and the likely existence of biased-signaling in terms of receptor/ligand interactions (8, 9), suggests that chemokine receptor involvement in inflammatory responses is complex and potentially redundant.

In the context of an inflammatory response, it is clear from a number of studies that numerous inflammatory chemokines are simultaneous expressed at damaged sites. These then attract leukocytes by interacting with inflammatory chemokine receptors and initiate inflammatory responses. While inflammation typically is transient, and resolves efficiently, occasionally, it can be associated with chronic inflammatory disease. The fact that chemokines and their receptors are the primary drivers of inflammatory leukocyte recruitment therefore highlights them as important therapeutic targets (10). Despite this exciting opportunity, progress toward development of clinically useful receptor antagonists has been extremely disappointing (11). Indeed, 25 years since the cloning of the first inflammatory chemokine receptor (12), there are still no chemokine receptor antagonists licensed for use in treating inflammatory diseases. While there are many pharmacological reasons for this, one over-riding reason is that we currently have a relatively poorly developed understanding of precisely how chemokines and their receptors orchestrate inflammatory responses and of the layers of complexity introduced as different inflammatory leukocytes enter, and exit, inflamed sites. Thus, a much more comprehensive understanding of this process is required for it to be effectively therapeutically targeted.

The resolution of the inflammatory response is a key step at which inflammation can transition, from an acute and transient response, to one that is chronic and pathological. Accordingly, there have been numerous studies into the molecular regulation of the resolution of the inflammatory response, which has highlighted lipid mediators, such as resolvins (13), as important regulators. In terms of removal of chemokines during resolution, this is achieved in two separate ways. First, most chemokines (and indeed other inflammatory cytokines) are removed from inflamed tissue by drainage through the lymphatic system (14). This almost certainly accounts for the high levels of inflammatory mediators and chemokines in the plasma of patients with chronic inflammatory pathologies. However, recent data have highlighted active roles for chemokine-scavenging atypical chemokine receptors (ACKRs) in the resolution of inflammatory responses (15). In this review, we discuss the roles for ACKRs in the resolution of the inflammatory response and highlight their potential therapeutic value.

## Atypical Chemokine Receptors

Atypical chemokine receptors (6, 16), (Table 1), in contrast to canonical chemokine receptors, are mainly expressed by non-leukocyte cell types, such as erythrocytes, lymphatic or vascular endothelial cells, although some expression of ACKRs (especially ACKR2 and ACKR3) is detected on leukocytes (6, 17, 18). ACKRs bind chemokines with high affinity and do not induce cell migration as a result of their structural inability to couple to G proteins. In fact, ACKR activation of β-arrestin-dependent pathways modulates chemokine bioavailability by transporting chemokines to intracellular degradative compartments or, in the case of polarized cells, to the opposite side of the cell monolayer (19). ACKRs can also modulate the chemokine system by regulating the expression, or signaling, of other canonical chemokine receptors (18).

**Table 1:** **Ligands and expression patterns for the ACKRs**.

Gene	Ligands	Expression
ACKR1	CCL2, 5, 7, 11, 13, 14, 17; CXCL5, 6, 8, 11	Erythrocytes, vascular endothelial cells, and Purkinje cells
ACKR2	CCL2, 3, 3L1, 4, 5, 7, 8, 11, 12, 13, 17, 22	Lymphatic endothelial cells, leukocytes (especially B1 B cells), keratinocytes, and trophoblasts
ACKR3	CXCL11, 12	Hematopoietic cells, lymphatic endothelial cells, mesenchymal cells, and neuronal cells
ACKR4	CCL19, 21, 25; CXCL13	Lymphatic endothelial cells and epithelial cells

Four molecules have been officially named and included in the ACKR subfamily: ACKR1, previously called duffy antigen receptor for chemokines (DARC); ACKR2, also known as D6 or CCBP2; ACKR3, also called CXC-chemokine receptor 7 (CXCR7) or RDC1; and ACKR4, previously called CC chemokine receptor-like 1 (CCRL1) and also known as CCX-CKR. Two other molecules, CCRL2 and PITPNM3, tentatively included in the ACKR family as “ACKR5” and “ACKR6,” respectively, will not be covered by this review as they are awaiting functional confirmation (16). It may be that additional ACKRs exist and these will be incorporated into the systematic nomenclature as they are identified (16). One of the problems in routinely identifying such receptors, for example, by de-orphanizing known orphan GPCRs is their lack of canonical signaling. Thus, each of the known atypical receptors has been identified through serendipity rather than through directed signaling-based screening approaches.

Here, we will review the involvement of the four characterized ACKRs in inflammation and its resolution.

## ACKR1 (DARC)

ACKR1 binds over 20 inflammatory chemokines belonging to the CC and CXC families (20). It is expressed by erythrocytes and endothelial cells lining small veins and venules (21). From a structural perspective, it is completely lacking the DRYLAIV motif in the second intracellular loop and has a low percentage of sequence homology with the other chemokine receptors (22). Thus, in contrast to the other ACKRs, the genes for which sit within chromosomal loci incorporating other canonical chemokine receptors, ACKR1 appears to share limited evolutionary relationship to the other receptors.

ACKR1 expressed by erythrocytes regulates the bioavailability of circulating chemokines by binding them with high affinity (23, 24). African people, referred to as “Duffy null” or negative because they lack ACKR1 expression on erythrocytes (but not endothelial cells), have higher concentrations of circulating chemokines (25), and genome-wide association studies have linked the ACKR1 variant Asp42Gly with serum CCL2 and CXCL8 levels (23). During inflammatory conditions, ACKR1 can function as a “sink” but also as a buffer for chemokines, increasing their systemic bioavailability and avoiding excessive changes in the concentration of circulating chemokines (26). In addition, it was found that ACKR1 expressed by endothelial cells is able to induce chemokine internalization and trancytosis (19, 27), thereby facilitating presentation of inflammatory chemokines on the luminal surface of vascular endothelial cells.

In the context of resolution of inflammation, the role of ACKR1 was studied in models of acute or chronic inflammation in WT and ACKR1 KO mice. Lack of the receptor results in reduced neutrophil recruitment to the lung after intratracheal administration of CXCL8 or LPS (28, 29) and in a model of acid-induced injury (30). Reduced neutrophil recruitment was also found in ACKR1 KO mice in a model of acute kidney damage induced by ischemia or LPS and was associated with renal protection (31). In a model of bone fracture, ACKR1 KO mice have decreased levels of pro-inflammatory cytokines (IL-1β, IL-6, and CCL2) and fewer macrophages around fractures (32). ACKR1 plays also a role in chronic inflammation, as demonstrated by the use of the ApoE KO mouse model of atherogenesis. ACKR1 KO mice are partially protected from atheroma development, and this is associated with decreased levels of inflammatory chemokines in the aorta and modest changes in T lymphocytes and inflammatory monocyte numbers in plaques (33). A role for ACKR1 was also found in infectious diseases: it is the receptor for the human malarial parasites *Plasmodium vivax* and *Plasmodium knowlesi* and individuals lacking ACKR1 (Duffy negative), or carrying polymorphic variants, are less susceptible to *P. vivax* infection (34).

The emerging picture is that ACKR1 expressed by erythrocytes acts as a chemokine buffer and can limit excessive leukocyte extravasation. In contrast, endothelial ACKR1 promotes acute and chronic inflammation by reducing chemokine concentrations in the inflamed tissues and creating a gradient that increases neutrophil and monocyte extravasation.

## ACKR2 (D6 or CCBP2)

ACKR2 is able to bind a broad panel of inflammatory CC chemokines. It is expressed by lymphatic endothelial cells, trophoblasts in the placenta, and some leukocytes such as alveolar macrophages and innate-like B cells (35). ACKR2 is a chemokine scavenger receptor which functions, in a catalytic manner, by transporting chemokines to degradative intracellular compartments (36, 37). It is able to dynamically adapt its scavenger function to the extracellular chemokine concentration activating a β-arrestin-dependent pathway that increases its plasma membrane localization without affecting the internalization rate (38, 39). ACKR2 promotes the resolution of inflammation and regulates lymphatic vessel function (40) and density (14), and ACKR2 KO mice in different pathological contexts exhibit dysregulated inflammatory reactions due to the lack of chemokine clearance and associated accumulation of inflammatory cells (41).

In response to phorbol ester, ACKR2 KO mice develop a severe skin inflammatory response resembling psoriasis (42), and after injection of complete Freund’s adjuvant, KO mice develop larger granulomas compared to WT mice (43). ACKR2 also controls inflammatory responses in the gut (44, 45) and in the lung (46). ACKR2 expressed by trophoblasts inhibits inflammation in the placenta, where it protects from inflammation-associated miscarriage and allogeneic embryo rejection (47, 48). After myocardial infarction, ACKR2 prevents excessive infiltration of classical monocytes and neutrophils by scavenging CCL2, promoting cardiac remodeling (49). ACKR2 is also important for the control of inflammation in infectious diseases such as *Mycobacterium tuberculosis* (50). The role of ACKR2 in the context of autoimmune diseases is still controversial. It was reported that ACKR2 KO are resistant to the induction of experimental autoimmune encephalomyelitis (EAE) (51) and have reduced renal inflammation in a model of diabetic nephropathy (52). More recently, it appears that ACKR2 deficiency does not suppress autoreactive T-cell priming and autoimmune pathology, but can enhance T-cell polarization toward Th17 cells (53).

In addition to these data indicating that ACKR2 promotes resolution of the inflammatory response by chemokine clearance and inhibition of excessive leukocyte recruitment, it was reported that ACKR2, expressed by leukocytes, inhibits their pro-inflammatory phenotype. ACKR2 restricts neutrophil migration (54) and regulates macrophage efferocytosis and cytokine secretion (55). Finally, a key role for ACKR2 in regulating the promotion of inflammation-dependent cancers has been shown using mouse models of both cutaneous (56) and colorectal cancer (45). In these contexts, ACKR2 functions essentially as a tumor suppressor gene by limiting tumor-promoting tissue inflammatory responses.

## ACKR3 (CXCR7 or RDC-1)

ACKR3 binds two chemokines, CXCL12, the ligand of CXCR4, and CXCL11, one of the ligands of CXCR3 (57). It is expressed by endothelial cells, some hematopoietic cells, mesenchymal cells, and neurons. ACKR3 mainly signals through β-arrestin pathways activating extracellular signal-regulated kinases (ERKs) or protein kinase B (PKB or Akt) (58). ACKR3 modulates CXCL12 activity in several ways. It downregulates CXCL12 concentrations by scavenging and modulates CXCR4 expression and signaling activity by forming heterodimers with CXCR4 (59). Elegant studies using zebrafish embryos have demonstrated important and evolutionary conserved roles for ACKR3 in the regulation of key cellular populations during embryogenesis (60). These studies have shed important light on the importance of ACKR3 for the generation of tissue gradients during cellular migration within the embryo. ACKR3 KO mice have defects in brain, heart, and kidney development (61–63).

During inflammatory conditions, both leukocytes and endothelial cells increase ACKR3 expression. Peripheral blood lymphocytes from patients with inflammatory bowel disease have enhanced ACKR3 expression, which was also upregulated upon stimulation (CD3) or costimulation (CD3/CD28) (64). ACKR3 was found expressed by macrophages in the atherosclerotic plaque and was associated with a pro-inflammatory phenotype that includes production of inflammatory chemokines and phagocytic activity (65). ACKR3 is also prominently expressed in a wide range of tumors both within the tumor cells and by cells of the tumor vasculature (66). It has therefore been highlighted as a potential therapeutic target in oncology.

In relation to endothelial cells, ACKR3 is expressed in rheumatoid arthritis synovium, in which it promotes the inflammatory process increasing angiogenesis (67). In addition, ACKR3 is induced in brain microvascular endothelial cells during experimental inflammatory conditions, such as permanent middle cerebral artery occlusion and EAE, and favors leukocyte extravasation by enhancing leukocyte adhesion to the endothelial surface (68). It should be noted that CXCR7 is also expressed by neurons and astrocytes in various brain regions and, during EAE, it is upregulated by oligodendrocyte progenitors, important cells for the remyelination process (69).

In summary, ACKR3 expression promotes inflammation inducing a leukocyte pro-inflammatory phenotype, enhancing angiogenesis and leukocyte extravasation.

## ACKR4 (CCRL1 or CCX-CKR)

ACKR4 binds the homeostatic chemokines CCL19, CCL21, CCL25, and CXCL13. It is expressed by thymic epithelial cells, bronchial cells, and keratinocytes. ACKR4 is a constitutively internalizing receptor with chemokine-scavenging function (70). After chemokine binding, it recruits β-arrestin 2, but it is not known if it activates signal transduction pathways.

Few data are available on the *in vivo* role of ACKR4 in the context of inflammation. It appears to be important in the correct trafficking of dendritic cells for the induction of adaptive immune responses. Indeed, ACKR4 expression in lymph nodes is necessary for creating a gradient of the CCR7 ligands, CCL19 and CCL21, in the subcapsular sinus (71). In addition, using ACKR4 in KO mice, it was demonstrated that homeostatic chemokine clearance is necessary to control excessive Th17 responses that can lead to immunopathologies (72).

## Concluding Remarks

The identification and characterization of the ACKRs has represented a major advance in our understanding of the overall orchestration of chemokine-driven immune and inflammatory responses. These receptors have been shown to play important roles in regulating cell migration in developmental, inflammatory, immune, and pathological contexts (Table 2). In this context, these receptors control the chemokine system by scavenging, transporting, or storing chemokines, but also by regulating the activity of canonical chemokine receptors with which they share the ligands by forming heterodimers or modulating their expression levels or signaling activity.

**Table 2:** **Phenotype of ACKRs knockout mice in inflammation and infection models**.

Gene deletion	Phenotype	Reference
ACKR1	Reduced neutrophil recruitment in acute inflammation models	(28–30)
	Renal protection in ischemia or LPS induced acute kidney damage	(31)
	Reduced macrophages infiltration in bone fracture model	(32)
	Reduced atheroma development in the Apo E KO	(33)
ACKR2	Severe skin inflammatory reaction similar to psoriasis	(42)
	Increased granulomatous inflammatory response	(43)
	Increased gut and lung inflammation	(44–46)
	Increased tissue damage after myocardial infarction	(49)
	Increased inflammation-associated miscarriage and allogeneic embryo rejection	(47, 48)
	Uncontrolled *Mycobacterium tuberculosis* infection	(50)
ACKR3^a^	Exacerbates neointimal hyperplasia	(65)
ACKR4	Excessive Th17 responses	(72)

*^a^Tamoxifen-inducible knockout*.

The essential roles that they play, particularly in the context of resolving inflammatory responses, highlights them as potential therapeutic targets. While the normal pharmacological approach is to develop chemokine receptor antagonists, in the case of the atypical receptors what would be more useful would be small molecule inducers of either expression or activity. Such inducers could work through known cytokine pathways that induce ACKRs (73) or by capitalizing on our developing understanding of the kinetics of cell surface mobilization of these receptors (37, 38).

If developed, these could be used to increase ACKR function and thus neutralize chemokine activity in a number of inflammatory pathologies. Topical application of such regulators could be envisaged as having therapeutic potential in, for example, psoriasis and intranasal administration in the context of lung inflammatory responses. Furthermore, it may be possible to adapt these molecules for use in cancer therapy to restrict cancer access to pro-tumorigenic inflammatory leukocytes.

Atypical chemokine receptors therefore represent novel therapeutic targets likely to benefit in a number of pathologies with unmet clinical need.

## Author Contributions

GG and RB wrote the text and approved the final submission.

## Conflict of Interest Statement

The authors declare that the research was conducted in the absence of any commercial or financial relationships that could be construed as a potential conflict of interest.
